# A recent overview on the biological and pharmacological activities of ferulic acid

**Published:** 2019-03-05

**Authors:** Jae Kwang Kim, Sang Un Park

**Affiliations:** 1 Division of Life Sciences and Convergence Research Center for Insect Vectors, Incheon National University, Incheon 22012, Korea; 2 Department of Crop Science, Chungnam National University, 99 Daehak-ro, Yuseong-gu, Daejeon, 34134, Korea

## ⁯⁯⁯

**Dear Editor,**

Ferulic acid (FA) is an important phenolic acid that is commonly present in the leaves, fruits, and seeds of most plants. Certain types of grasses, including rice, wheat, and oats, are highly concentrated sources of FA. The name, ferulic, originates from the genus, *Ferula*, referring to giant fennel (*Ferula communis*). The International Union of Pure and Applied Chemistry (IUPAC) name for FA is (*E*)-3-(4-hydroxy-3-methoxy-phenyl) prop-2-enoic acid (Srinivasan et al., 2007[40]; Bento-Silva et al., 2018[4]). In plants, FA is biosynthesized from caffeic acid by the enzyme caffeate O-methyltransferase. FA, along with dihydroferulic acid, acts as a component of lignocellulose, which crosslinks lignins and polysaccharides, thereby conferring rigidity to the cell walls (de Oliveira et al., 2015[11]).

FA has been recognized as an important chemical structure serving several biological activities, including antioxidant, anti-inflammatory, antiviral, antiallergic, antimicrobial, antithrombotic, anticarcinogenic, and hepatoprotective actions, directly or indirectly (Kumar and Pruthi, 2014[23]; Mancuso and Santangelo, 2014[25]). The FA enrichment in different food items could reduce oxidative damage and amyloid pathology, especially for Alzheimer disease (Nabavi et al., 2015[29]; Sgarbossa et al., 2015[37]). In this review, we summarize the recent findings on the biological and pharmacological activities of FA (Table 1(Tab. 1); References in Table 1: Asadpour et al., 2018[1]; Aswar and Patil, 2016[2]; Bami et al., 2017[3]; Canturk, 2018[5]; Chen et al., 2018[6]; Cheng et al., 2016[7]; Chowdhury et al., 2016[8]; Colonnello et al., 2018[9]; Das et al., 2016[10]; Eitsuka et al., 2016[12]; El-Ashmawy et al., 2018[13]; Fong et al., 2016[14]; Gerin et al., 2016[15]; Gong et al., 2017[16]; Gu et al., 2017[17]; Hahn et al., 2016[18]; Hassanzadeh et al., 2017[19]; Hassanzadeh et al., 2018[20]; Ibitoye and Ajiboye, 2018[21]; Jayamani et al., 2018[22]; Macías-Cruz et al., 2018[24]; Maruyama et al., 2018[26]; Mir et al., 2018[27]; Mu et al., 2018[28]; Nagai et al., 2017[30]; Park et al., 2018[31]; Perez-Ternero et al., 2017[32]; Qi et al., 2017[33]; Sadar et al., 2016[34]; Sagar et al., 2016[35]; Salazar-López et al., 2017[36]; Shao et al., 2018[38]; Sompong et al., 2017[39]; Sudhagar et al., 2018[41]; Szulc-Kielbik et al., 2017[42]; Wang et al., 2017[43]; Yang et al., 2016[44]; Yu et al., 2016[45]; Yuan et al., 2016[46]; Zeni et al., 2017[47]; Zhang et al., 2018[48]; Zhou et al., 2017[50]; Zhou et al., 2018[49]).

*EXCLI Journal 2019;18:132-138 – ISSN 1611-2156*
*Received: February 07, 2019, accepted: February 26, 2019, published: March 05, 2019*

| **Key findings** | **Reference** |
|---|---|
| In a recent study, it was reported that FA improves hepatic fibrosis through the nhibition of the transforming growth factor (TGF)-β1/*Smad* pathway *in vitro* and *in vivo*. These findings revealed that FA can potentially be used to protect against liver fibrosis. | Mu et al., 2018 |
| FA enhances the antibacterial activity of quinolone-based antibiotics against *Acinetobacter baumannii* by enhancing reactive oxygen species (ROS) generation, energy metabolism, and the activity of the electron transport chain with a concomitant decrease in glutathione | Ibitoye and Ajiboye, 2018 |
| Chen et al. reported that FA can potentially treat various disorders, including NG-nitro-l-arginine methyl ester (L-NAME) induced preeclampsia in rats by decreasing placental inflammation and apoptosis. | Chen et al., 2018 |
| Research findings revealed that FA fights against kidney ischemia reperfusion injury by decreasing apoptosis, increasing adenosine generation, reducing inflammation, and upregulating CD39 and CD73 expression. | Zhou et al., 2018 |
| Supplementation of FA in other foods or ingredients enriches the development of the reproductive tract and ovarian activity of pre-pubertal hair breed ewe lambs during the natural anestrous season. An enhancement in the functionality of the glucose-insulin system could be a cause of this beneficial effect of FA. | Macías-Cruz et al., 2018 |
| FA and sugarcane aqueous extract (SCAE) can protect against toxic conditions. All of these effects are not necessarily related to SCAE, because FA requires the skn-1 pathway to exert its protective manner in *Caenorhabditis elegans*. | Colonnello et al., 2018 |
| FA intervention significantly ameliorates human umbilical vein endothelial cells (HUVEC) radiation injury through the thrombomodulin pathway. Therefore, FA could be effectively used  as a potential agent to attenuate radiation-induced damage. | Shao et al., 2018 |
| FA performs better than caffeic acid as an inhibitor of melanin production; the differences in the inhibitory efficacy of these two substances might be attributed to the difference in their tyrosine-binding activity. This study reveals that both substances effectively inhibited the CK2 (casein kinase 2)-mediated phosphorylation of tyrosinase. | Maruyama et al., 2018 |
| FA and fish oil (FO) demonstrate anti-inflammatory and renoprotective effects through their peroxisome proliferator-activated receptor gamma (PPAR-γ) agonistic activity. Both FA and FO are natural products, and they both can offer a safe intervention strategy after exposure to nephrotoxins. | El-Ashmawy et al., 2018 |
| FA is considered as a remedy for the plaques related to collagen deposition, because it is a potential inhibitor of collagen fibrillation and its propagation. | Jayamani et al., 2018 |
| FA has protective effects against lipopolysaccharide (LPS)-induced acute kidney injury (AKI) in mice, which might suggest a chemopotential role treating AKI in humans. | Mir et al., 2018 |
| FA, along with caspofungin, has synergistic effects against *Candida albicans*. These two compounds help to combine the existing anticandidal drug with phytochemicals to increase the efficacy of the anticandidal drug. | Canturk, 2018 |
| FA and quercetin exhibit excellent bioavailability and bioactivity against some metabolic syndromes, like inflammatory bowel diseases and obesity. | Zhang et al., 2018 |
| The new poly(ether ester urethane)urea elastomer (PEEUU) adapted with FA could act as a promising candidate for the vascular application of enhancing blood compatibility and vascular cell-compatibility. | Asadpour et al., 2018 |
| FA mixed with nanostructured lipid carriers (NLCs) improves the pharmacological profile of FA and activates the phosphatidylinositol 3-kinase (PI3K) pathway, which has a therapeutic value against cerebral stroke. | Hassanzadeh et al., 2018 |

| Key findings | Reference |
|---|---|
| FA stimulates the synthesis of procollagen and hyaluronic acid, the inhibition of metalloproteinase, and the reduction in matrix metalloproteinase (MMP)-1 and MMP-9 expression in CCD-986sk cells stimulated with ultraviolet B (UV-B). FA can potentially be used as functional food for whitening and anti-wrinkle activities. | Park et al., 2018 |
| FA combined with-vinylguaiacol forms a chemical starting structure for the development of new small molecules that protects against epidermal growth factor receptor (EGFR). | Sudhagar et al., 2018 |
| FA has endothelium-independent vascular relaxant responses in different types of arteries. The molecular mechanism of FA-induced vasorelaxation involves the inhibition of a calcium channel and calcium desensitization. | Zhou et al., 2017 |
| FA has beneficial effects on diabetes-induced cognition lesions, which was revealed by the regulation of the protein tyrosine phosphatase 1B (PTP1B) and insulin signaling pathways. PTP1B inhibition may be an approach to remedy abnormal signaling linked to diabetes-induced cognitive impairment. | Wang et al., 2017 |
| FA shows potential therapeutic efficacy in enhancing survival and differentiation of neural stem cells (NSCs) to protect against gentamicin-induced neuronal hearing loss. | Gu et al., 2017 |
| FA shows antiepileptogenic effects and protects against oxidative stress and cognitive impairment induced by pentylenetetrazol kindling by acting as a promising adjuvant for antiepileptic drugs. | Hassanzadeh et al., 2017 |
| FA acts as an anti-inflammatory and antioxidant agent on macrophages due to its free radical scavenging activity in a cell free system. Consuming FA in a diet can defend the host from the development and/or progression of inflammation. | Szulc-Kielbik et al., 2017 |
| In rice bran, FA represents an active component that enhances the expression of mitochondrial biogenesis and dynamics markers. In a vascular damage mouse model, FA decreases oxidative stress in endothelial cells and human mononuclear cells. | Perez-Ternero et al., 2017 |
| FA could be considered as a novel agent to increase the management of depression, because it repairs stress caused by the hypothalamic-pituitary-adrenal-axis dysfunction. | Zeni et al., 2017 |
| FA treatment significantly protects against oxidative stress, shows positive antioxidative activity, and improves histological parameters to normal, exhibiting the nephroprotective and antioxidant effects of this phenolic compound. | Bami et al., 2017 |
| FA enhances the obesogenic status induced by a high-fat diet (HFD), and the integral effects of FA on a biological system were elucidated. | Salazar-López et al., 2017 |
| FA reduces preeclampsia symptoms in a rat preeclampsia model, exhibiting its potential value in treating preeclampsia. | Gong et al., 2017 |
| FA diminishes the increase in gene expression and assembly of proteins related to the emission of three types of A$\beta$ peptides in $H_2O_2$-stimulated human lens epithelial (HLE) cells. These findings provide evidence of the antioxidative functions of FA in lens epithelial cells. | Nagai et al., 2017 |
| The oxygen-carrying capacity of a hemoglobin site specifically adapted with ferulic acid (FA-Hb) was similar to endogenous Hb, but the rate of autoxidation of FA-Hb was much lower than Hb in various systems. | Qi et al., 2017 |
| Pretreated with 0.1 mM of FA impairs the methylglyoxal (MG)-induced decrease of cell viability and protects against MG-induced cell apoptosis in pancreatic β-cells. These findings suggest that FA is capable of protecting β-cells from MG-induced cell damage in diabetes. | Sompong et al., 2017 |
| FA shows protective effects against ultraviolet A (UVA)-induced cell damage through antioxidant and stress-inducible cellular action in human dermal fibroblasts (HDFs). | Hahn et al., 2016 |
| FA significantly improved the intracellular concentration of δ-tocotrienol (T3), enhancing the bioavailability of δ-T3, and thus increasing the inhibitory power of δ-T3 on telomerase. For the above mentioned activities, FA could be a promising candidate to target δ-T3 and augment the anti-cancer activity. | Eitsuka et al., 2016 |

| Key findings | Reference |
|---|---|
| FA could effectively act against acetaminophen-induced liver injury by down-regulating the expression of CYP 2E1 and the inhibition of Toll-like receptor (TLR) 4-mediated inflammatory responses. | Yuan et al., 2016 |
| FA could inhibit the interferon-γ (IFN-γ)-induced inflammatory response by reducing the release of pro-inflammatory cytokines to improve trinitrobenzenesulfonic acid-induced colitis. | Sadar et al., 2016 |
| FA protects the initiation of apoptotic signaling in the spleen by obstructing the free radical chain reaction and by scavenging superfluous ROS. FA can prevent the spleen from ionizing radiation. | Das et al., 2016 |
| FA has a potential therapeutic response exhibiting antioxidant and hypoglycemic effects, which might help in circumventing stress-mediated diabetic cardiomyopathy in rats. | Chowdhury et al., 2016 |
| FA is a promising candidate to treat developmental lead neurotoxicity. These hopeful findings will initiate future investigations evaluating the FA-mediated potentiation of neurite outgrowth following lead exposure *in vivo*. | Yu et al., 2016 |
| *Trans*-FA at concentrations between 0.06 to 0.6 mM shows anti-proliferation and anti-migration effects in the human lung cancer cell line, H1299. | Fong et al., 2016 |
| FA prevents osteoclast fusion by reducing the expression of dendritic cell-specific transmembrane protein (DC-STAMP) and enhancing apoptosis in mature osteoclasts through the caspase-3 pathway. | Sagar et al., 2016 |
| FA-loaded hydrogel (thermosensitive) could salvage Cisd2-deficient (Cisd2(-/-)) cardiomyocytes (CM) from oxidative stress-induced damage and could acted as a potential therapeutic in the future treatment of cardiovascular diseases (CVD). | Cheng et al., 2016 |
| The anti-hyperalgesia response of FA, which might be related to its antioxidant and anti-inflammatory activity, in rats with chronic constriction injury (CCI) could be effective as an adjuvant to conventional medicines. FA is also related to the protection of neuropathic pain. | Aswar and Patil, 2016 |
| A study by Yang et al. shows that FA significantly inhibits important diseases, such as d-galactose(d-gal)-induced AchE (acetylcholinesterase) activity, neuroinflammation and neurodegeneration, and oxidative stress, thus consequently ameliorates memory impairment. | Yang et al., 2016 |
| FA is a promising hepatoprotective agent against formaldehyde toxicity, because it exhibits positive effects on oxidative stress parameters. | Gerin et al., 2016 |

## *Conflict of interest*

The authors declare no conflict of interest.

## Acknowledgements

This research was supported by Golden Seed Project (213006051WTE11) funded by Ministry of Agriculture, Food and Rural Affairs (MAFRA), Ministry of Oceans and Fisheries (MOF), Rural Development Administration (RDA) and Korea Forest Service (KFS), Republic of Korea.

## Conflict of interest

The authors declare no conflict of interest.

## Figures and Tables

**Table 1:**
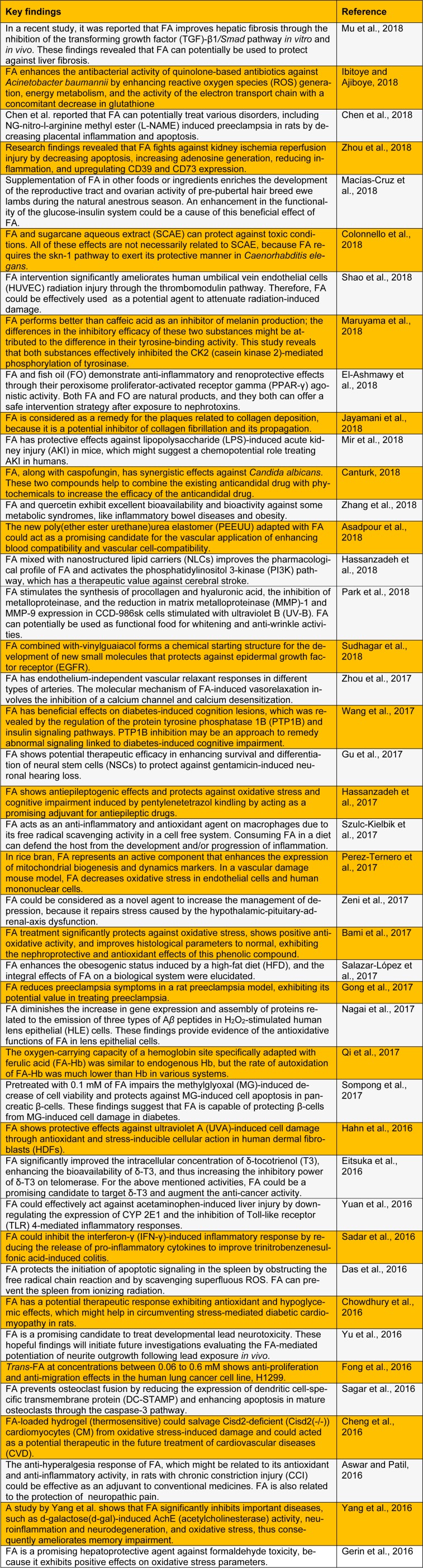
Recent studies on the biological and pharmacological activities of ferulic acid

## References

[1] Asadpour S, Ai J, Davoudi P, Ghorbani M, Jalali Monfared M, Ghanbari H (2018). In vitro physical and biological characterization of biodegradable elastic polyurethane containing ferulic acid for small-caliber vascular grafts. Biomed Mater.

[2] Aswar M, Patil V (2016). Ferulic acid ameliorates chronic constriction injury induced painful neuropathy in rats. Inflammopharmacology.

[3] Bami E, Ozakpınar OB, Ozdemir-Kumral ZN, Köroglu K, Ercan F, Cirakli Z (2017). Protective effect of ferulic acid on cisplatin induced nephrotoxicity in rats. Environ Toxicol Pharmacol.

[4] Bento-Silva A, Vaz Patto MC, do Rosário Bronze M (2018). Relevance, structure and analysis of ferulic acid in maize cell walls. Food Chem.

[5] Canturk Z (2018). Evaluation of synergistic anticandidal and apoptotic effects of ferulic acid and caspofungin against Candida albicans. J Food Drug Anal.

[6] Chen Y, Xue F, Han C, Yang H, Han L, Li K (2018). Ferulic acid ameliorated placental inflammation and apoptosis in rat with preeclampsia. Clin Exp Hypertens.

[7] Cheng YH, Lin FH, Wang CY, Hsiao CY, Chen HC, Kuo HY (2016). Recovery of oxidative stress-induced damage in Cisd2-deficient cardiomyocytes by sustained release of ferulic acid from injectable hydrogel. Biomaterials.

[8] Chowdhury S, Ghosh S, Rashid K, Sil PC (2016). Deciphering the role of ferulic acid against streptozotocin-induced cellular stress in the cardiac tissue of diabetic rats. Food Chem Toxicol.

[9] Colonnello A, Kotlar I, de Lima ME, Ortíz-Plata A, García-Contreras R, Soares FAA (2018). Comparing the effects of ferulic acid and sugarcane aqueous extract in in vitro and in vivo neurotoxic models. Neurotox Res.

[10] Das U, Biswas S, Sengupta A, Manna K, Chakraborty A, Dey S (2016). Ferulic acid (FA) abrogates ionizing radiation-induced oxidative damage in murine spleen. Int J Radiat Biol.

[11] de Oliveira DM, Finger-Teixeira A, Mota TR, Salvador VH, Moreira-Vilar FC, Molinari HB (2015). Ferulic acid: a key component in grass lignocellulose recalcitrance to hydrolysis. Plant Biotechnol J.

[12] Eitsuka T, Tatewaki N, Nishida H, Nakagawa K, Miyazawa T (2016). A combination of δ-tocotrienol and ferulic acid synergistically inhibits telomerase activity in DLD-1 human colorectal adenocarcinoma cells. J Nutr Sci Vitaminol (Tokyo).

[13] El-Ashmawy NE, Khedr NF, El-Bahrawy HA, Helal SA (2018). Upregulation of PPAR-γ mediates the renoprotective effect of omega-3 PUFA and ferulic acid in gentamicin-intoxicated rats. Biomed Pharmacother.

[14] Fong Y, Tang CC, Hu HT, Fang HY, Chen BH, Wu CY (2016). Inhibitory effect of trans-ferulic acid on proliferation and migration of human lung cancer cells accompanied with increased endogenous reactive oxygen species and β-catenin instability. Chin Med.

[15] Gerin F, Erman H, Erboga M, Sener U, Yilmaz A, Seyhan H, Gurel A (2016). The effects of ferulic acid against oxidative stress and inflammation in formaldehyde-induced hepatotoxicity. Inflammation.

[16] Gong W, Wan J, Yuan Q, Man Q, Zhang X (2017). Ferulic acid alleviates symptoms of preeclampsia in rats by upregulating vascular endothelial growth factor. Clin Exp Pharmacol Physiol.

[17] Gu L, Cui X, Wei W, Yang J, Li X (2017). Ferulic acid promotes survival and differentiation of neural stem cells to prevent gentamicin-induced neuronal hearing loss. Exp Cell Res.

[18] Hahn HJ, Kim KB, Bae S, Choi BG, An S, Ahn KJ (2016). Pretreatment of ferulic acid protects human dermal fibroblasts against ultraviolet A irradiation. Ann Dermatol.

[19] Hassanzadeh P, Arbabi E, Atyabi F, Dinarvand R (2017). Ferulic acid exhibits antiepileptogenic effect and prevents oxidative stress and cognitive impairment in the kindling model of epilepsy. Life Sci.

[20] Hassanzadeh P, Arbabi E, Atyabi F, Dinarvand R (2018). Ferulic acid-loaded nanostructured lipid carriers: A promising nanoformulation against the ischemic neural injuries. Life Sci.

[21] Ibitoye OB, Ajiboye TO (2018). Ferulic acid potentiates the antibacterial activity of quinolone-based antibiotics against Acinetobacter baumannii. Microb Pathog.

[22] Jayamani J, Naisini A, Madhan B, Shanmugam G (2018). Ferulic acid, a natural phenolic compound, as a potential inhibitor for collagen fibril formation and its propagation. Int J Biol Macromol.

[23] Kumar N, Pruthi V (2014). Potential applications of ferulic acid from natural sources. Biotechnol Rep (Amst).

[24] Macías-Cruz U, Vicente-Pérez R, López-Baca MA, González-Ríos H, Correa-Calderón A, Arechiga CF (2018). Effects of dietary ferulic acid on reproductive function and metabolism of pre-pubertal hairbreed ewes during the anestrous season. Theriogenology.

[25] Mancuso C, Santangelo R (2014). Ferulic acid: pharmacological and toxicological aspects. Food Chem Toxicol.

[26] Maruyama H, Kawakami F, Lwin TT, Imai M, Shamsa F (2018). Biochemical characterization of ferulic acid and caffeic acid which effectively inhibit melanin synthesis via different mechanisms in B16 melanoma cells. Biol Pharm Bull.

[27] Mir SM, Ravuri HG, Pradhan RK, Narra S, Kumar JM, Kuncha M (2018). Ferulic acid protects lipopolysaccharide-induced acute kidney injury by suppressing inflammatory events and upregulating antioxidant defenses in Balb/c mice. Biomed Pharmacother.

[28] Mu M, Zuo S, Wu RM, Deng KS, Lu S, Zhu JJ (2018). Ferulic acid attenuates liver fibrosis and hepatic stellate cell activation via inhibition of TGF-β/Smad signaling pathway. Drug Des Devel Ther.

[29] Nabavi SF, Devi KP, Malar DS, Sureda A, Daglia M, Nabavi SM (2015). Ferulic acid and Alzheimer's disease: promises and pitfalls. Mini Rev Med Chem.

[30] Nagai N, Kotani S, Mano Y, Ueno A, Ito Y, Kitaba T (2017). Ferulic acid suppresses amyloid β production in the human lens epithelial cell stimulated with hydrogen peroxide. Biomed Res Int.

[31] Park HJ, Cho JH, Hong SH, Kim DH, Jung HY, Kang IK (2018). Whitening and anti-wrinkle activities of ferulic acid isolated from Tetragonia tetragonioides in B16F10 melanoma and CCD-986sk fibroblast cells. J Nat Med.

[32] Perez-Ternero C, Werner CM, Nickel AG, Herrera MD, Motilva MJ, Böhm M (2017). Ferulic acid, a bioactive component of rice bran, improves oxidative stress and mitochondrial biogenesis and dynamics in mice and in human mononuclear cells. J Nutr Biochem.

[33] Qi D, Li Q, Wang P, Wang X (2017). Haemoglobin site-specifically modified with ferulic acid to suppress the autoxidation. Artif Cells Nanomed Biotechnol.

[34] Sadar SS, Vyawahare NS, Bodhankar SL (2016). Ferulic acid ameliorates TNBS-induced ulcerative colitis through modulation of cytokines, oxidative stress, iNOs, COX-2, and apoptosis in laboratory rats. EXCLI J.

[35] Sagar T, Rantlha M, Kruger MC, Coetzee M, Deepak V (2016). Ferulic acid impairs osteoclast fusion and exacerbates survival of mature osteoclasts. Cytotechnology.

[36] Salazar-López NJ, Astiazarán-García H, González-Aguilar GA, Loarca-Piña G, Ezquerra-Brauer JM, Domínguez Avila JA (2017). Ferulic acid on glucose dysregulation, dyslipidemia, and inflammation in diet-induced obese rats: an integrated study. Nutrients.

[37] Sgarbossa A, Giacomazza D, di Carlo M (2015). Ferulic acid: a hope for Alzheimer's disease therapy from plants. Nutrients.

[38] Shao S, Gao Y, Liu J, Tian M, Gou Q, Su X (2018). Ferulic acid mitigates radiation injury in human umbilical vein endothelial cells in vitro via the thrombomodulin pathway. Radiat Res.

[39] Sompong W, Cheng H, Adisakwattana S (2017). Ferulic acid prevents methylglyoxal-induced protein glycation, DNA damage, and apoptosis in pancreatic β-cells. J Physiol Biochem.

[40] Srinivasan M, Sudheer AR, Menon VP (2007). Ferulic acid: therapeutic potential through its antioxidant property. J Clin Biochem Nutr.

[41] Sudhagar S, Sathya S, Anuradha R, Gokulapriya G, Geetharani Y, Lakshmi BS (2018). Inhibition of epidermal growth factor receptor by ferulic acid and 4-vinylguaiacol in human breast cancer cells. Biotechnol Lett.

[42] Szulc-Kielbik I, Kielbik M, Klink M (2017). Ferulic acid but not alpha-lipoic acid effectively protects THP-1-derived macrophages from oxidant and pro-inflammatory response to LPS. Immunopharmacol Immunotoxicol.

[43] Wang H, Sun X, Zhang N, Ji Z, Ma Z, Fu Q (2017). Ferulic acid attenuates diabetes-induced cognitive impairment in rats via regulation of PTP1B and insulin signaling pathway. Physiol Behav.

[44] Yang H, Qu Z, Zhang J, Huo L, Gao J, Gao W (2016). Ferulic acid ameliorates memory impairment in d-galactose-induced aging mouse model. Int J Food Sci Nutr.

[45] Yu CL, Zhao XM, Niu YC (2016). Ferulic acid protects against lead acetate-induced inhibition of neurite outgrowth by upregulating HO-1 in PC12 cells: involvement of ERK1/2-Nrf2 pathway. Mol Neurobiol.

[46] Yuan J, Ge K, Mu J, Rong J, Zhang L, Wang B (2016). Ferulic acid attenuated acetaminophen-induced hepatotoxicity though down-regulating the cytochrome P 2E1 and inhibiting toll-like receptor 4 signaling-mediated inflammation in mice. Am J Transl Res.

[47] Zeni ALB, Camargo A, Dalmagro AP (2017). Ferulic acid reverses depression-like behavior and oxidative stress induced by chronic corticosterone treatment in mice. Steroids.

[48] Zhang L, Dong M, Xu G, Tian Y, Tang H, Wang Y (2018). Metabolomics reveals that dietary ferulic acid and quercetin modulate metabolic homeostasis in Rats. J Agric Food Chem.

[49] Zhou Q, Gong X, Kuang G, Jiang R, Xie T, Tie H (2018). Ferulic acid protected from kidney ischemia reperfusion injury in mice: possible mechanism through increasing adenosine generation via HIF-1α. Inflammation.

[50] Zhou ZY, Xu JQ, Zhao WR, Chen XL, Jin Y, Tang N (2017). Ferulic acid relaxed rat aortic, small mesenteric and coronary arteries by blocking voltage-gated calcium channel and calcium desensitization via dephosphorylation of ERK1/2 and MYPT1. Eur J Pharmacol.

